# Do family physicians perceive electronic cigarette use as a harm reduction strategy for smokers? A survey from Istanbul

**DOI:** 10.1017/S1463423622000056

**Published:** 2022-03-21

**Authors:** Ozlem Tanriover, Seyhan Hidiroglu, Pinar Ay, Robert L. Cook

**Affiliations:** 1 Department of Family Medicine and Medical Education, Yeditepe University, Faculty of Medicine, Istanbul, Turkey; 2 Department of Public Health, Marmara University, School of Medicine, Istanbul, Turkey; 3 Department of Epidemiology, University of Florida, Gainesville, FL, USA

**Keywords:** e-cigarettes, family physician, harm reduction, nicotine addiction, smoking cessation

## Abstract

**Background:**

E-cigarettes (ECs) are gaining popularity in Turkey among smokers. With the rapid increase of EC consumption, it is important to ascertain how family physicians (FPs) perceive ECs as they play a key role in providing smoking cessation services.

**Aim:**

Our main objectives were to determine FPs’ level of awareness and harm reduction perceptions of ECs and to delineate the factors associated with their counseling practices.

**Methods:**

This was a cross-sectional study with descriptive and analytical components. Data were collected through questionnaires. Questions mainly focused on demographic characteristics, knowledge and own use of ECs, general attitudes towards ECs, and daily practices while performing counseling on tobacco use. In order to control confounding factors, logistic regression analysis was performed.

**Findings:**

Among a total of 271 FPs, 49.1% (*n* = 133) were males and the median age (IQR) was 41 years (32–46). Almost one-fifth of the FPs (*n* = 52) reported that they did not provide counseling services to their smoking patients. Only 26.6% (*n* = 72) of the FPs stated that they felt confident enough to advise patients on smoking cessation. Of the FPs, 6.6% have stated that they have recommended ECs to their patients for smoking cessation with the strategy of harm reduction. Factors associated with providers’ recommendation of ECs to their patients as a harm reduction strategy included ‘believing that ECs help smokers to quit, ECs could be vaped in closed areas, and ECs were healthier than combustible tobacco products’.

**Conclusion:**

In our study, FPs stated lack of confidence to advice patients on smoking cessation. Furthermore, they recommended ECs to their smoking patients as a harm reduction strategy. FPs’ confidence should be increased with the trainings based on recent evidence on ECs.

## Introduction

Turkey has had a long tradition of tobacco use and a high smoking prevalence. Being a tobacco-producing country, nearly one-third of Turkey’s adults continue to use tobacco being the subject of a notorious expression ‘smoking like a Turk’ (TurkStat, 2019). E-cigarettes (EC) are gaining popularity in Turkey among smokers (Goney *et al*., 2019).

In Turkey, ECs are not legal and are not legally sold, and furthermore, the Turkish Ministry of Health has not approved them as a smoking cessation method. However, many people procure ECs and e-liquids through online distributors or bring them from other countries.

ECs have been debated extensively, and they have not been approved as a tobacco cessation aid by medical authorities (Gualano *et al*., 2015; Hartmann-Boyce *et al*., 2016; Khoudigian *et al*., 2016; Malas *et al*., 2016). Moreover, recent research indicates adverse health consequences of EC use. Mc Connell *et al.* documented that adolescent EC users had increased rates of chronic bronchitis symptoms (McConnell *et al*., 2017). In addition to that Bahl *et al*. showed that EC refill fluids have cytotoxic effects (Bahl *et al*., 2012). These refill fluids contained toxicants such as diacetyl and diethylene glycol (Westenberger *et al*., 2009; Varlet *et al*., 2015; Allen *et al*., 2016), and EC aerosols contained formaldehyde-hemiacetals, ultrafine particles, and metals (Williams *et al*., 2013; Jensen *et al.*, 2015) which caused DNA strand breaks and reduced cell survival in vitro (Yu *et al*., 2016) and the aldehydes can cause lung and cardiovascular disease (Ogunwale *et al*., 2017). Furthermore, ECs contain acrolein, which can cause acute lung injury and COPD and may cause asthma and lung cancer (Bein and Leikauf, 2011). Recently, toxic potential of vaping is reported causing exogenous lipoid pneumonia and diffuse alveolar hemorrhage with proven alveolar injury, as well as vaping-associated bronchiolitis obliterans (Petrella, 2021).

Despite the negative health effects, the prevalence of EC use and its popularity has increased rapidly throughout the years. In a systematic review of forty-four studies from Europe and North America, the frequency of those who were aware of ECs increased from 16% in 2009 to 58% in 2011, while EC use increased from 1% to 6% (Pepper *et al*., 2014). Similarly, the prevalence of lifetime EC use reported among adults in the United States rose from 0.6% in 2009 to 4.9% in 2018 (King *et al*., 2015, Gentzke *et al*., 2019), and in another study from the United States, ever use of EC increased from 1.8% in 2010 to 13.0% in 2013, while current use increased from 0.3% to 6.8% (McMillen *et al*., 2015). Recent studies show that 22%–30% of young adults in United States report lifetime EC use and 5%–9% are current EC users (Johnston *et al*., 2014; Delnevo *et al*., 2016). This high prevalence of ever EC use is in line with previous studies indicating that the popularity of EC use among young adults has increased rapidly (Sutfin *et al*., 2013; Littleﬁeld *et al.*, 2015; Saddleson *et al*., 2015).

Smoking or nicotine addiction is defined as a disease in the International Classification of Diseases by WHO (World Health Organization, 1993). For this reason, treatment of smoking dependence is regarded as a responsibility of the physician (Piné-Abata *et al.*, 2013). FPs are key actors for smoking cessation counseling. However, this high prevalence of ECs that are promoted as smoke-quit devices adds some challenge to the clinician’s role in directing patients’ smoking cessation efforts. ECs are relatively new products and are not currently recommended in the guidelines of Turkish Ministry of Health. Furthermore, they are not recommended by organizations like the World Health Organization and the Food and Drug Administration (Schier *et al*., 2019; Layden *et al.*, 2020).

Current research indicates that a majority of physicians report discomfort talking to their patients about ECs due to the limited knowledge (Geletko *et al.*, 2016; Vasconcelos and Gilbert, 2019). Furthermore, until clinical guidelines are set, FPs will encounter questions about ECs from their patients. In several studies, it was shown that physicians were believing that ECs were safer alternatives to smoking combustible tobacco products and they showed willingness to support their patients’ desire to use ECs (El-Shahawy *et al.*, 2016). In another study, Kandra *et al.* indicated that increased odds of recommending ECs to patients is associated with physicians who believed ECs lower the risk of cancer for patients who use them instead of smoking cigarettes, increased frequency of patient inquiry about ECs, older physicians, and those physicians who documented tobacco use counseling in their clinic notes (Kandra *et al*., 2014). Therefore, it is important to find out the perceptions of FPs about these products when giving counseling to their smoking patients. There is limited evidence in the literature about FPs’ awareness, knowledge, and counseling practices regarding ECs, and there is no study reported from Turkey on this subject (Kandra *et al.*, 2014; Pepper *et al*., 2015; Drouin *et al*., 2016; El-Shahawy *et al*., 2016; Geletko *et al.*, 2016; Lazuras et al., 2016; Moysidou *et al*., 2016; Van Gucht and Baeyens, 2016; Egnot *et al.*, 2017; Nickels *et al.*, 2017; Ofei-Dodoo *et al.*, 2017, Mughal *et al*., 2018). Hence, our main objectives were to determine FPs’ level of awareness and harm reduction perceptions of ECs and to delineate the factors associated with their counseling practices.

## Methods

### Design, sample, and setting

This is a cross-sectional study with descriptive and analytical components. Our aim was to determine the prevalence of family physicians recommending e-cig to their patients. In order to reveal the prevalence, we used a cross-sectional design.

A total of 3 districts of Istanbul serve as the research and training area of the Marmara University School of Medicine. These areas receive migrations from mainly southern and eastern regions of Turkey. Therefore, the population of these districts have a potential to resemble Turkey’s overall population. Family physicians working in Family Health Centers in the mentioned districts constituted the research population. A total of 401 family physicians work, 161 in Ümraniye, 123 in Maltepe, and 117 in Kartal Districts. In these districts, a total of 98 family health centers serve as the primary care facilities for the population. When recommending EC use as a harm reduction strategy was assumed to be 5% among FPs, with 95% confidence level and 1.5% margin of error the sample size was calculated as 270. However, for practical reasons, we wanted to encompass all of the family physicians working in the area.

Data were collected by self-administered questionnaires. The questionnaire was prepared by the study group based on relevant literature. The questionnaires were distributed to the study group in their work settings by Public Health research assistants organized by one of the research members (SH). SH invited all of the FPs to participate in the study after receiving their informed consent.

A self-administered questionnaire was developed based on literature review and pilot tested on FPs working in an academic setting. After the pilot survey, we asked for comments on any unclear or awkward questions and revised the survey according to the feedback.

SH and research assistants left the questionnaires to the FPs in their work settings requesting them to fill out the questionnaire and collected them at the end of the day. As these districts serve as the research and training area of the Marmara University School of Medicine, the FPs comply very well with such questionnaires.

Questions mainly focused on demographic characteristics, knowledge and self-use of ECs, general attitudes, and counseling practices regarding EC use. The questionnaire was consisted of 10 questions regarding demographic characteristics, 5 questions on knowledge and self-use of ECs, 6 questions on general attitudes, 15 questions on counseling practices regarding EC use.

A written informed consent was obtained from each participant before administering the questionnaire.

**The main dependent variables were as follows:** the knowledge, attitudes, and behaviors of FPs about ECs and their advice/recommendation to their patients/clients. In addition to that EC counseling and counseling features were also evaluated. (Their daily practices while performing counseling on tobacco cessation). The questionnaire focused on physician perceptions of recommending ECs for tobacco cessation.

**The independent variables were as follows:** sociodemographic variables, such as age, gender, date of graduation from medical school, average number of patients seen per week, smoking status, participation in smoking cessation training.

### Data analysis

Data were analyzed using the SPSS software, version 21(SPSS Inc., Chicago, Illinois, USA). Descriptive variables were evaluated with frequency, mean ± SD, median, and percentiles. Odds ratios (ORs) were calculated for assessing the strength of associations. ORs were calculated for 2 analyses: (1) The Association of Family Physicians Perception of Competence and Behavior on Smoking Cessation, the Idea of EC Harm Reduction and Their Recommendations of ECs to Their Patients, in Univariate Analyses; and (2) Factors associated with family physicians’ recommendation of ECs to their patients as a harm reduction strategy, in multivariate analyses

Confidence intervals of 95% for prevalence and ORs were determined. Categorical variables were compared through the chi-square and Fisher’s tests. Logistic regression analysis was used to identify variables that were independently associated with recommendation e-cigarettes as a harm reduction strategy. Statistical significance was defined as *P* < 0.05.

Among the providers who recommend EC use to their patients, we have grouped the FPs using ‘harm reduction strategy’ as (1) those who recommend ECs as an alternative to quit smoking for their patients, (*n* = 13, % 4.8, 95% CI: 2.7–8.1); (2) those who recommend to continue to use ECs to their patients who were already using ECs to quit smoking, (*n* = 29, 10.7% 95% CI: 7.5–14.9); and (3) those who recommend to continue to use ECs in closed areas to their patients who do not want to quit smoking (*n* = 28, 10.3%, 95% CI:7.2–14.5).

### Ethical approval

This study was approved by the Ethical Board of Marmara University – Institute of Health Sciences, Istanbul Turkey.

### Findings

A total of 271 FPs participated in the study (response rate: 67.6 % (271/401). Of the participants, 49.1% (*n* = 133) were male and the median age was 41 years. The mean number of years after graduation was 15.88 ± 2.57.

Among all, 50.2% (*n* = 136) were never smokers, 24. 7% (*n* = 67) were former smokers, 11.8% (*n* = 32) were current smokers with intention to quit, and 8.1% (*n* = 22) were current smokers with no intention to quit. Of the respondents, 17. 7% (*n* = 48) had taken training on tobacco and smoking cessation.

Table 1 presents FPs’ awareness and their recommendation of ECs to their patients. Among all, 86% (*n* = 233) have ever heard of ECs. Of the FPs, 6.6% (*n* = 18) reported that they had recommended ECs to their patients for smoking cessation.

**Table 1.** Awareness and recommendation of e-cigarettes by FPs

	Yes *n* (%)		95% CI
Have you ever heard of e-cigarettes?	233	(86.0)	81.3–89.6
Have you ever seen e-cigarette ads?	122	(45.0)	39.2–50.9
Have you ever seen e-cigarette sales?	75	(27.7)	22.6–33.2
Have you ever recommended e-cigarettes to your patients?	18	(6.6)	4.1–10.3

Only 26.6% (*n* = 72) of the FPs (95% CI: 21.6–32.1) stated that they felt confident enough to advise patients on smoking cessation and 16.2% (*n* = 44) (95% CI: 12.3–21.1) felt confident about writing appropriate prescriptions for smoking cessation (Table 2).

**Table 2.** FPs’ clinical practices and feeling of confidence of their own efficacy to help their patients quit smoking

	Yes *n* (%)		95% CI
I always ask my patients if they currently smoke.	253	(93.4)	89.6–95.8
I always ask my patients if they have smoked before.	200	(73.8)	68.2–78.6
I always ask my patients how much and what type of cigarettes they smoke.	145	(53.5)	47.5–59.3
I feel confident to counsel my patients about smoking cessation.	72	(26.6)	21.6–32.1
I give efficient counseling to my patients for smoking cessation.	64	(23.6)	18.9–29.0
I feel confident enough to write a proper prescription for smoking cessation.	44	(16.2)	12.3–21.1
I write proper prescription for smoking cessation.	29	(10.7)	7.5–14.9

Harm reduction perceptions of FPs’ about ECs and their clinical practices are presented in Table 3. Of the FPs, 22.5% (*n* = 61) thought that ECs were healthier than combustible tobacco products. Approximately ten percent of FPs stated that they suggested that patients should continue to smoke ECs who were already using them or wanted to smoke in enclosed spaces. Among all, 17.7% (*n* = 48) believed that risk of cancer was lower among EC users compared to traditional cigarette smokers.

**Table 3.** Harm reduction perceptions of FPs about e-cigarettes and their clinical practices

Statements	Agree *n* (%)	
Harm reduction perceptions		
E-cigarettes are healthier than combustible tobacco products.	61	22.5
E-cigarettes have an incentive effect on children and adolescents.	172	63.5
Smoke from e-cigarettes is safe.	37	13.7
E-cigarette smokers have a lower risk of cancer.	48	17.7
E-cigarettes can be vaped in closed areas.	35	12.9
E-cigarettes work for people who want to quit smoking.	39	14.4
Clinical practices		
I recommend e-cigarettes to my patients as an alternative method to quit smoking.	13	4.8
I recommend my patients to continue to use e-cigarettes in closed areas who do not want to quit smoking.	28	10.3
I recommend my patients to continue to vape e-cigarettes who are already using them to quit smoking.	29	10.7

The factors associated with FPs’ recommendations of ECs to their patients were evaluated using univariate analyses. There was no association between gender, smoking status, participation in smoking cessation training, and recommendations of ECs. When ≤50 years of age were taken as the reference category, being >50 years increased the recommendation of ECs as a way of harm reduction strategy (OR 2.72; 95% CI: 1.08–6.85).

The association of FPs’ perceptions of competence on smoking cessation and their recommendation of ECs to their patients (univariate analyses) is shown in Table 4. The odds of recommendation of ECs to their patients were higher among those who stated that they gave adequate counseling to quit smoking (OR 2.49; 95% CI: 1.28–4.85) and who stated that they were writing appropriate prescriptions for smoking cessation to their patients (OR 2.77; 95% CI: 1.19–6.44).

**Table 4.** The association of FPs’ perception of competence on smoking cessation, and their recommendations of e-cigarettes to their patients with the idea of harm reduction, univariate analyses

	OR	95% CI	*P* value
I feel confident to counsel my patients about smoking cessation.	2.49	1.28–4.85	0.007
I give efficient counseling to my patients for smoking cessation.	2.11	1.07–4.17	0.03
I feel confident enough to write a proper prescription for smoking cessation.	2.32	1.10–4.90	0.02
I write proper prescription for smoking cessation.	2.77	1.19–6.44	0.01
E-cigarettes help smokers to quit.	14.47	6.62–31.60	*P* < 0.001
E-cigarettes can be vaped in closed areas.	9.82	4.50–21.45	*P* < 0.001
E-cigarettes are healthier than combustible tobacco products.	8.08	4.05–16.12	*P* < 0.001
E-cigarette smokers have a lower risk of cancer compared to conventional cigarette smokers	7.16	3.53–14.55	*P* < 0.001
The vapor (smoke) of e-cigarettes is safe.	6.56	3.08–13.98	*P* < 0.001
E-cigarettes have an incentive effect on children and adolescents.	0.93	0.48–1.80	0.836

Factors associated with FPs’ recommendation of ECs to their patients as a harm reduction strategy (multivariate analyses) are presented in Table 5. Factors associated with providers recommendation of ECs to their patients as a harm reduction strategy included FPs who believed that ECs help smokers to quit (OR 7.0, 95% CI: 2.82–17.50) among those who thought that ECs could be vaped in closed areas (OR 3.4, 95% CI: 1.26–9.25) among those who thought that ECs were healthier than combustible tobacco products (OR 3.13, 95% CI: 1.31–7.49) and among those who thought that they were writing proper prescription for smoking cessation (OR 2.97, 95% CI: 1.00–8.81).

**Table 5.** Factors associated with FPs’ recommendation of e-cigarettes to their patients as a harm reduction strategy, multivariate analyses^*^

	OR	95% CI	*P* value
I am writing proper prescription for smoking cessation.	2.97	1.00–8.81	0.05
E-cigarettes are healthier than combustible tobacco products.	3.13	1.31–7.49	0.010
E-cigarettes can be vaped in closed areas.	3.42	1.26–9.25	0.015
E-cigarettes help smokers to quit.	7.03	2.82–17.50	*P* < 0.001

* Variables analyzed: FPs’ Statements: ‘I give efficient counseling to my patients for smoking cessation’. ‘I am writing proper prescription for smoking cessation’. ‘E-cigarettes help smokers to quit’. ‘E-cigarettes can be vaped in closed areas’. ‘E-cigarettes are healthier than combustible tobacco products’. ‘E-cigarette smokers have a lower risk of cancer’. ‘The vapor (smoke) of e-cigarettes is safe’. ‘E-cigarettes have an incentive effect on children and adolescents’.

In our study, almost one-fifth of the FPs (*n* = 52) reported that they did not provide counseling services to their smoking patients (Table 6). Moreover, almost 60% (*n* = 126) stated that they did not record the patients after they have given smoking cessation counseling.

**Table 6.** Opinions of FPs on smoking cessation counseling services

	*n*	(%)
I advise my patients to quit smoking		
To all of them	69	(25.8)
To most of them	83	(31.1)
To half of them	18	(6.7)
Less than half	45	(16.9)
To none	52	(19.5)
I record my patients after I have provided counseling on smoking cessation^*^		
All of them	21	(9.9)
Most of them	26	(12.3)
Half of them	6	(2.8)
Less than half	33	(15.6)
None	126	(59.4)
I have recommended my patients to use electronic cigarettes for smoking cessation		
Yes	18	(6.6)
No	253	(93.4)

* Only those who counsel their patients to quit smoking were included in this analysis.

## Discussion

### Summary of findings

To the best of our knowledge, this is the first study to examine Turkish FPs’ harm reduction strategy in recommending ECs to their smoking patients. We have found out that almost one-fifth of the FPs (*n* = 52) reported that they did not provide counseling services to their smoking patients. Furthermore, only 26.6% (*n* = 72) of the FPs stated that they felt confident enough to advise patients on smoking cessation.

We have also demonstrated that FPs recommended ECs to their patients if they perceived that ECs were healthier than combustible tobacco products. Our findings also suggest that in our sample while it was relatively a small number of FPs recommended ECs to their patients, it was noteworthy that they perceived EC use as a harm reduction strategy.

Physicians can contribute to tobacco control in a variety of ways. These include being a role model (non-smoking); giving recommendations to quit smoking; providing smoking cessation treatment; and helping to reshape public policies in order to control the consumption of tobacco products (National Institute for Health and Clinical Excellence, 2008). Therefore, finding out the key elements of clinical practice of FPs in smoking cessation is of vital importance.

In recent years, the number of scientific research regarding ECs has increased exponentially, leading to ongoing debate, but the main finding is that in comparison with some other quit methods, the number of supportive articles is very limited (Heydari *et al.*, 2017). However, ECs are seen as effective strategies by the public for harm reduction and smoking cessation (Etter and Bullen, 2014; Barakat *et al*., 2021). One of our research questions was that whether FPs shared the same views or not. It appears that using ECs to quit smoking is one method considered by some patients who seek to quit smoking, although there is no evidence that they are effective in helping to reduce smoking.

In the multivariate analysis, we have demonstrated that FPs recommended ECs to their patients if they perceived that ECs helped smokers to quit and they could be vaped in closed areas and if they thought that ECs were healthier than combustible tobacco products. The majority of the FPs was not aware that ECs have not been proven to be effective, reliable, and safe methods to help with quitting cigarettes. Our findings suggest that some of the FPs perceive EC use as a harm reduction strategy when recommending ECs to their patients.

### Strength and limitations

To our knowledge, this is the first study to examine Turkish FPs’ attitudes and behaviors on harm reduction strategy in recommending ECs to their smoking patients. This study also serves as a needs assessment approach for FPs who need up-to-date trainings based on evidence on smoking cessation including newer products like ECs.

A number of methodological limitations must be considered in interpreting our findings in this study. First, this study was carried out only with a relatively small number of physicians working in three provinces of Istanbul. Second, data were not based on observation of the behavior of FPs in particular, and the data were self-reported and subjective. The third is that the questionnaire forms have been filled in by two-thirds of the FPs and the findings in non-participants may be different. Therefore, the generalization of the findings of this study may be limited, but it is important to emphasize that FPs need more training on ECs.

### Comparison with existing literature

El-Shahawy *et al.* and Ferrara *et al.* found that most primary care physicians thought that ECs were safer alternatives to combustible cigarettes, and they recommended ECs as a safer alternative to smoking combustible tobacco products (El-Shahawy *et al.*, 2016; Ferrara *et al*., 2019). It is clear that primary care physicians in their study were also using a harm reduction strategy when recommending ECs to their patients. Similarly, Kandra et al stated that two-thirds of the physicians participating in their study (included 156 FPs, 161 internal medicine doctors, 159 obstetricians, 160 psychiatrists, and 151 surgeons; 787 physicians in total from US) reported that ECs helped people to quit smoking and 35% offered ECs to their patients. Factors influencing physicians’ recommendations for patients include increased frequency of patient inquiry about ECs, and physicians feel that ECs are safer than standard cigarettes (Kandra *et al.*, 2014).

Another area where physicians can contribute to smoking control is to recommend their patients to quit smoking. Such advice should include a clear request to quit, reinforcing personal risks of smoking and their reversibility, offering solutions to barriers to quitting, and offering treatment. All smokers should be encouraged to use both medications and counseling. According to previous studies, the intention of the patients to quit smoking (Yao *et al.*, 2009) was related to the doctor’s recommendation. In another study, 40% of smokers attempted to quit with a doctor’s recommendation (Kreuter *et al.*, 2000). As a result of a meta-analysis, 26 000 smokers indicated that with a very brief doctors’ recommendation quit rates would significantly increase (Kottke *et al*., 1988; Lancaster *et al*., 2000). This is why each physician should ask all patients whether they smoke or not, advise them to quit smoking (Fiore *et al.*, 2014), and use the recommended strategies that take very little time. In our study, nearly one-fifth of the FPs did not consider giving counseling to any smokers and 15.9% of FPs stated that they did not ask their patients whether they were smoking cigarettes. Only 26.6% (*n* = 72) of FPs felt confident enough to advise on smoking cessation, and even fewer (16.2%, *n* = 44) were confident that they could provide appropriate prescriptions for smoking cessation. One of the interesting findings of our study was the relationship between FPs’ perceptions of competence and behavior on smoking cessation and their recommendations of ECs to their patients. The ORs of recommendation of ECs to their patients was higher among those who also stated that they were writing appropriate prescriptions for smoking cessation to their patients. This result may suggest that physicians who are confident in counseling their patients in smoking cessation perceive ECs as nicotine replacement therapy and advise their patients to use them as harm reduction strategy.

## Conclusions (implications for research and practice)

To our knowledge, this study is the first to examine the level of awareness of ECs, the perceptions of harm reduction, and the factors affecting clinical practice of FPs in Turkey. Our findings show that further studies are needed to explore more deeply the factors that influence the level of knowledge and clinical practice on smoking cessation and in particular of EC counseling. Testing the harm vs benefits of ECs would be a useful subject of future inquiries. Moreover, in our study few physicians felt confident about managing smoking demonstrates a need for large national efforts across the country to disseminate preventive programs on combating tobacco in Turkey.

In our study, FPs stated lack of confidence to advice patients on smoking cessation. Furthermore, some of them recommended ECs to their smoking patients as a harm reduction strategy. Family physicians’ confidence should be increased with the trainings based on recent evidence on ECs.
